# Complete Traumatic Trifocal Failure of the Extensor Mechanism of the Knee: A Case Report and Review of the Literature

**DOI:** 10.1155/2019/4695282

**Published:** 2019-11-11

**Authors:** Alexander J. Johnson, Katharine D. Harper, Christopher Haydel

**Affiliations:** Temple University Hospital Department of Orthopaedics and Sports Medicine, USA

## Abstract

The unique case of a rare 3-level extensor mechanism failure in a 28-year-old male, involving a tibial tubercle avulsion fracture, a patellar tendon avulsion off the tibial tubercle fragment, and a severely comminuted patella fracture, and the surgical technique required to repair such an injury is presented. Focus is spent on the unique repair of a tendon injury when both proximal and distal bony attachments are damaged. Trifocal knee extensor mechanism is a rare clinical entity with minimal literature available—to date, this injury has only been reported in a retrospective review of combat-related injuries in military personnel. It is important to maintain an understanding of knee extensor mechanism anatomy and perform thorough investigation of high-energy knee injuries to ensure adequate treatment of all injuries. The outcome presented in this case shows that positive results after complex extensor mechanism injuries may be achieved, but limited data exists to elucidate optimum treatment. It is essential for surgeons to have firm grasp of techniques used to treat each segment of the extensor mechanism so that they may be combined when a patient presents with complex, multifocal injury.

## 1. Introduction

Traumatic extensor mechanism injuries are common, especially in those under 40 years of age. Most commonly, when the extensor mechanism fails, the failure occurs at a single location along the chain, with patella fractures being more common than other locations. [1] This paper will discuss in detail the unique case of a 3-level extensor mechanism failure and the surgical technique required to repair such an injury. The patient presented had a comminuted patellar fracture, a patellar tendon avulsion off the tibial tubercle, and a tibial tubercle fracture. Focus will be spent on the unique repair of a tendon injury when both proximal and distal bony attachments are damaged.

## 2. Case Details

**Figure 1:**
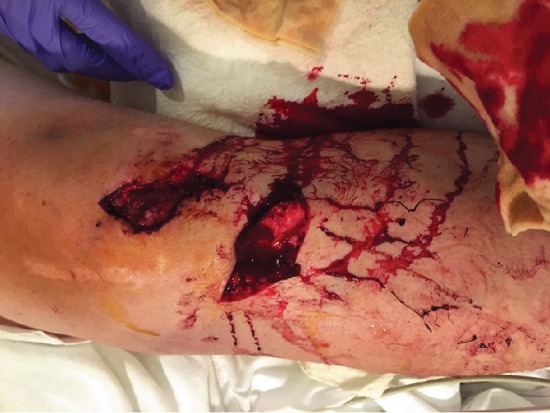
Picture demonstrating the wounds sustained in the initial injury, with underlying exposed bone of the fracture site.

**Figure 2:**
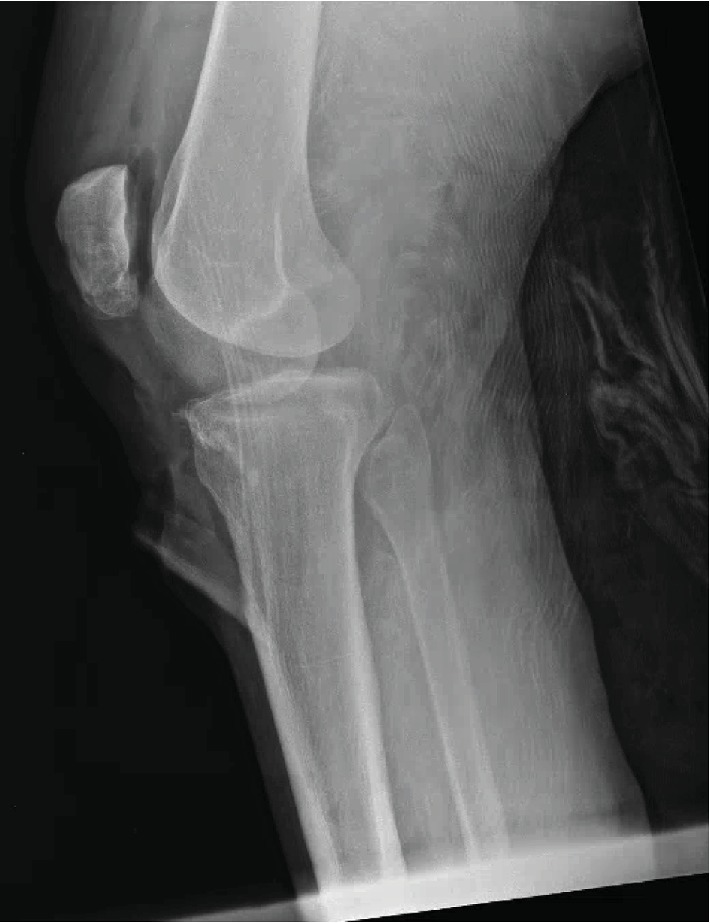
Lateral radiograph demonstrating tibial tubercle fracture and comminuted patellar fracture with overlying soft tissue defects.

**Figure 3:**
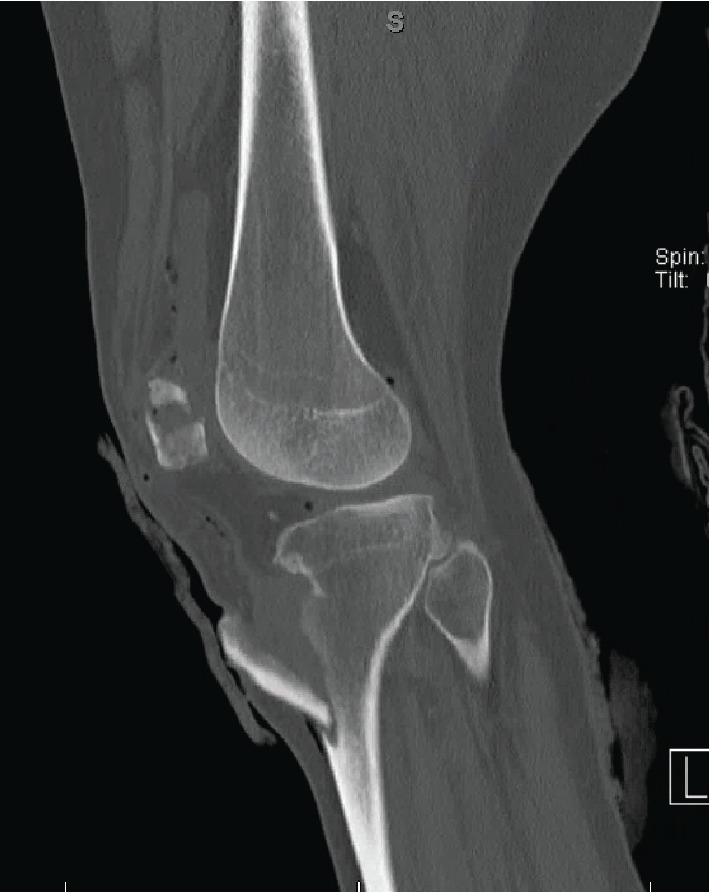
CT scan delineating the vertical, coronal plane fracture line of the extra-articular tibial tubercle fracture with overlying soft tissue injury. Also demonstrates a large bony defect in the patella, confirming the comminute nature of the fracture.

A 28-year-old male presented to the emergency department after a motor vehicle accident (MVC). The patient had a body mass index (BMI) of 36, no significant past medical history, and no history of smoking. There was a possible history of remote, pediatric “knee surgery,” but this history could not be confirmed by the patient. He was driving an 18-wheeler truck in the accident, which had the front end of the cab collapse into the cabin. At time of presentation to the trauma bay, he was initially assessed by the trauma service via Advanced Trauma Life Support (ATLS) standards and was determined to be hemodynamically stable with an isolated orthopaedic injury to his left lower extremity. The left lower extremity had two visible approximately 5 cm wounds over the anterior knee (Figure 1). Initial radiographs showed a tibial tubercle fracture and comminuted patella fracture (Figure 2). Computerized tomography (CT) scan was acquired that showed a vertical fracture line in the coronal plane of the tibial tubercle and comminuted patellar fracture (Figure 3). The patient received 2 g cefazolin in the emergency room for prophylaxis for his open fracture, as well as a tetanus vaccine. He was then emergently brought to the operating room for irrigation and debridement of his open fracture wounds and ORIF for his patella and tibia. In the operating room, the injury was classified as a Gustilo-Anderson Grade II open tibia fracture with associated Gustilo-Anderson Grade II open patella fracture by the operative attending based on the wounds each measuring < 10 cm with evidence of vascular injury and no need for soft tissue coverage procedure.

## 3. Surgical Technique

**Figure 4:**
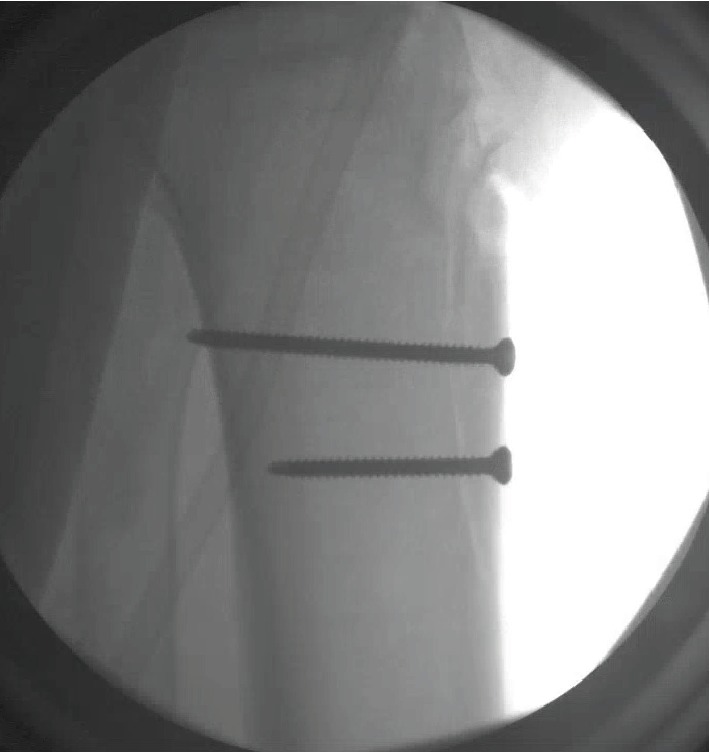
Intraoperative fluoroscopic images confirming anatomic reduction of the tibial tubercle fragment with 4.0 cannulated bicortical screws, placed in a lag screw fashion.

There were two large wounds, one overlying the anterior tibia (with visualized tubercle fracture) as well as one overlying the patella (Figure 1). The distal-most, transverse wound was extended distally to improve visualization of the underlying fracture. These wounds were initially explored, and the patellar tendon was identified and mobilized from the overlying and underlying tissue. Once the tendon was mobilized, it was discovered to have avulsed from its tibial tubercle insertion. The most distal wound was extended distally along the lateral aspect of the tibial crest for complete visualization of the fracture. The fracture was mobilized and debrided of soft tissue, periosteum, and hematoma. After debridement, initial irrigation of the open wound was performed with 3L NS by low-pressure gravity tubing. Following that, an anatomical reduction was achieved on the fracture fragment and K-wire placement performed for initial fixation. Following that, two Synthes 3.5 mm cortical lag screws (Synthes DePuy; Warsaw, IN) were placed in the proximal and distal aspect of the fracture fragment. Adequate compression was achieved at the fracture site. Screw placement was confirmed under fluoroscopy to be in adequate position (Figure 4).

**Figure 5:**
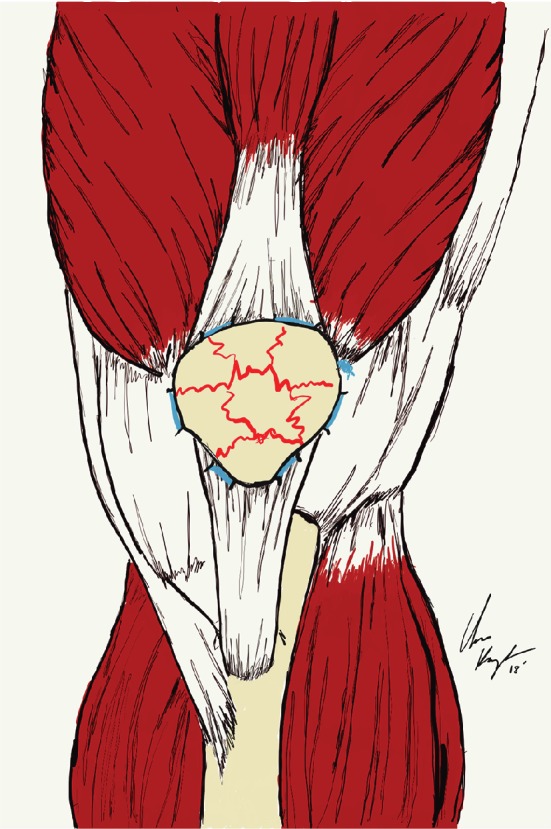
An illustration demonstrating the unique, purse-string technique employed for fixation of the patella fracture with overlying soft tissue deficit.

Attention was then turned to the patellar fracture. Patellar retinaculum was investigated and determined to be intact, with a comminuted patella fracture underlying with minimal displacement of fragments. A small retinacular window was created to observe the largest fragment, and a point-to-point bone clamp was placed on the medial and lateral aspect of the patella to compress the fracture fragments. Following compression, a #2 fibre wire was placed in a cerclage, purse-string fashion around the patellar retinacular soft tissue and hand tied under tension (Figure 5). Clamp was removed and good compression across comminuted fragments was found with gross alignment maintained. The patella was then irrigated with 3L NS solution, and the retinacular window was closed using #0 vicryl suture in an interrupted fashion.

**Figure 6:**
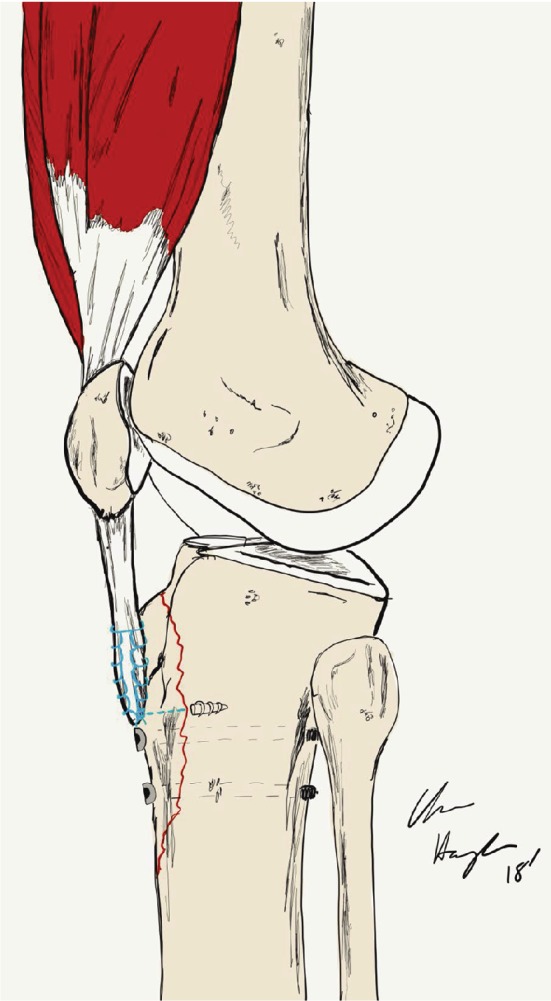
An illustration demonstrating the placement of anchor suture drill holes for fixation of the patellar tendon insertion, bypassing previously fixated fracture site.

**Figure 7:**
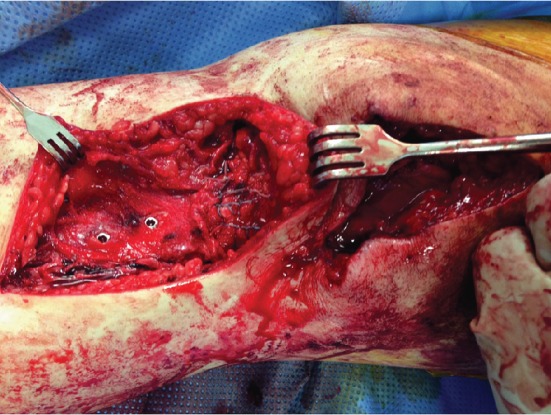
Intraoperative clinical picture showing the completed repair of the tibial tubercle fracture, patellar fracture, and patellar tendon avulsion.

Finally, the patellar tendon insertion avulsion was addressed. Due to the associated tibial tubercle fracture, this presented a unique challenge in acquiring adequate fixation. Two Arthrex 5.5 mm corkscrew anchor sutures (Arthrex; Naples, FL) were placed at the tibial insertion just medial to and through the previously fixed fracture fragment. The anchors were advanced from anterior to posterior through the fracture fragment and into a stable tibial bone (Figure 6). The anchors were tested manually and found to be well fixed in a stable bone. Attached #2 fibre wire was then stitched through the patellar tendon in a Krakow stitch using a free needle. Two complete distal to proximal then proximal to distal rows of sutures were performed and tied overtop the tendon. The insertion site was then reinforced with #0 vicryl suture. The entire extensor mechanism repair was tested under flexion without evidence of gapping at the tibial tubercle fracture site, patellar fracture site, or patellar tendon repair site (Figure 7).

**Figure 8:**
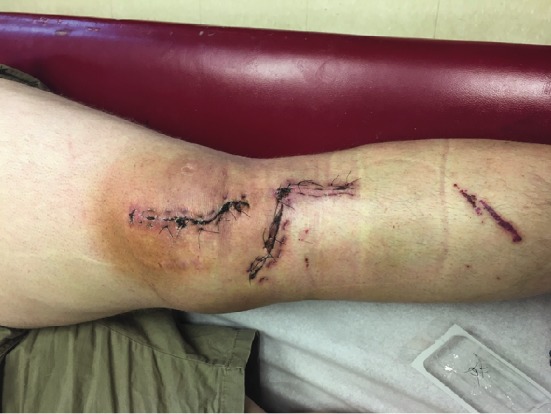
Clinical photograph at first follow-up visit showing well-healing wounds without evidence of skin necrosis, wound breakdown, erythema, or drainage.

**Figure 9:**
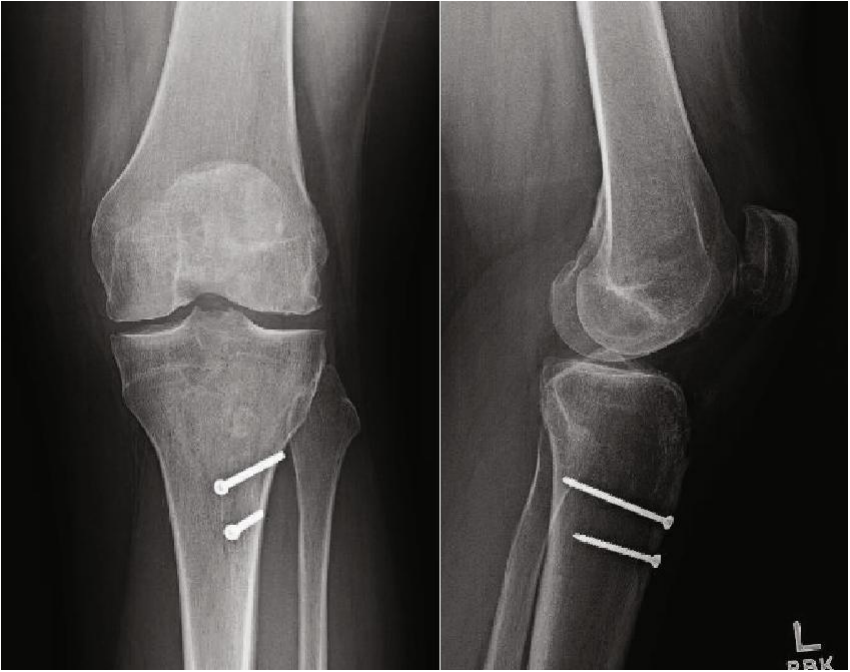
AP and lateral radiograph taken at 1-year follow-up showing healed fracture and no evidence of hardware complications.

Following surgery, the patient was hospitalized for 24 hours of antibiotic treatment, compartment monitoring, and pain control. He was discharged with partial weight bearing in a hinged knee brace locked in extension on postoperative day 1. First follow-up visit occurred 10 days after surgery and showed well-healing wounds without evidence of skin necrosis (Figure 8). Final follow-up occurred at 1 year postsurgery; his wounds have healed without issue; he has complete bony union of all fractures, has returned to full activities, and has no complaints at this time. X-rays from final follow-up are shown in Figure 9.

## 4. Discussion

We have described a case of trifocal knee extensor mechanism failure including a tibial tubercle avulsion fracture, a patellar tendon avulsion off the tibial tubercle fragment, and a severely comminuted patella fracture. Failure of the knee extensor mechanism in three locations is exceedingly rare with the only identified report in a retrospective review of combat-related injuries in military personnel [1]. In that series, 17 reviewed complex open extensor mechanism injuries were caused by explosive devices in 15 cases and high energy gunshots in 2 cases compared to the current case which involved a restrained tractor trailer driver in a high-speed motor vehicle collision. The authors proposed a novel classification scheme based on the segment of the extensor mechanism that was injured, soft tissue status, and associated distal femur or proximal tibia fracture requiring fixation. According to this classification, the injury in the current case would be labeled a 345A0, an injury combination that was not present in any of the 17 reviewed cases. The series did include 4 cases of trifocal extensor mechanism failure and 1 case of four-level failure.

In adults, patellar tendon avulsions/ruptures are more common in patients with elevated body mass index (BMI), underlying systemic illness, or steroid use [2]. The patient in the current case did have an elevated BMI of 36, but did not clearly possess any other risk factors, although no dedicated workup of systemic illness was performed. Generally, patellar avulsion and tendinous failure of the extensor mechanism occur via force applied across a flexed knee, while comminuted or open patella fractures occur via direct trauma [2]. The injury in the current case is most likely related to a combined mechanism evidenced by the tubercle avulsion fracture and the comminuted patella fracture with overlying open wound. At 10-month follow-up, our patient has minimal pain over the anterior knee, intact extensor mechanism with 5 out of 5 knee extension strength, and range of motion of 0 to 130 degrees at the knee. He was able to return to activities of daily living and employment as a truck driver with frequent requirements to lift heavy objects and has returned to weight training in the gym, reporting focus on squat exercises. The authors consider this patient to have a better than expected outcome given the extent of injury. In one previous outcome study, there was a trend for patients with open patella fractures to have more associated injuries, higher pain scores, lower functional outcome scores, and higher incidence of complications, though this data did not reach statistical significance [3].

In limited case series, patella fractures were open in 7-13% of cases and were treated with antibiotics, incision, and debridement and various forms of fracture fixation with or without internal fixation [3–5]. In the current case, the patient was treated with immediate antibiotics and incision and debridement. The fracture was treated with purse-string closure of the overlying periosteum without internal fixation because the highly comminuted fracture had limited fixation options and high risk of infection existed given large open wound. While the previously cited series reported infection rates between 0 and 10.7% [3–5], there was no evidence of infection in the current case.

Although examples of trifocal extensor mechanism failure are lacking in nonmilitary series, there are a number of reports of bifocal failure. Kang et al. summarized these reports and presented a case of bifocal knee extensor mechanism disruption in an 84-year-old male after a motorcycle accident resulting in an open avulsion fracture of the inferior patellar pole and avulsion fracture of the tibial tubercle [6]. In this case, the patellar avulsion was repaired with three nonabsorbable number 2.0 Ethibond sutures using a vertical wiring technique [7], and the tibial tubercle avulsion was repaired with a 4.0 cannulated screw. The authors also proposed a classification system for double disruptions of the knee extensor mechanism. The injury presented in the current case resembles a type 1 injury under this classification system—avulsion fracture of tibial tubercle with patellar ligament avulsion off the tibial tubercle—with an ipsilateral comminuted patella fracture. In the literature review presented by Kang et al., 14 reported cases of type 1 injuries were identified, all in patients 18 years or younger, further illustrating the rarity of the case presented here in a 28-year-old patient [6].

Though reports of such injuries are limited in adult literature, several repair techniques have been described in pediatric cases of combined tibial tubercle and patella tendon avulsion injuries. Common to most of these reported techniques is open reduction and internal fixation of the tubercle fragment with screw fixation, but K-wire and staple fixation have also been described [6]. Multiple techniques for repairing the patella tendon injury have been described including staple fixation of the patellar tendon to the tibia distal to the tubercle fracture [8], suture fixation of the patellar tendon through transverse transosseous tunnels in the tibia distal to the tubercle fragment [9], and suture repair of the patella tendon to the tibial periosteum distal to the tubercle fracture [10]. Regardless of the method used, the repair of the avulsed tendon is commonly performed with fixation distal to the reduced tibial tubercle fragment, as it was in this case.

## 5. Conclusion

Although the injury described in this case does appear to be extremely rare, it is important to maintain a complete understanding of knee extensor mechanism anatomy and perform thorough investigation of high-energy knee injuries to ensure adequate treatment and improved outcomes in injuries that could be otherwise devastating to future function. The case and technique reviewed in this case demonstrate that acceptable outcomes after complex extensor mechanism injuries are possible, but limited data exists to elucidate optimum treatment. It is essential for surgeons who may encounter similar injuries to have firm grasp of techniques used to treat injury to each segment of the extensor mechanism so that they may be combined accordingly when a patient presents with complex, multifocal injury.
